# Clinical Evidence Regarding the Dynamic of Baker Cyst Dimensions after Intermittent Vacuum Therapy as Rehabilitation Treatment in Patients with Knee Osteoarthritis

**DOI:** 10.3390/jcm12206605

**Published:** 2023-10-18

**Authors:** Elena-Valentina Ionescu, Liliana-Elena Stanciu, Andreea Bujduveanu, Mihaela Minea, Doinita Oprea, Adina Petcu, Madalina-Gabriela Iliescu, Viorela-Mihaela Ciortea, Florina-Ligia Popa, Emma Gheorghe, Bogdan Obada, Carmen Oprea

**Affiliations:** 1 Balneal and Rehabilitation Sanatorium of Techirghiol, 34-40, Dr. Victor Climescu Street, 906100 Techirghiol, Romania; elena.ionescu@365.univ-ovidius.ro (E.-V.I.); liliana.stanciu@365.univ-ovidius.ro (L.-E.S.); andreea.bujduveanu@sbtghiol.ro (A.B.); mihaelamihaela.minea@sbtghiol.ro (M.M.); doinita.oprea@365.univ-ovidius.ro (D.O.); carmen.oprea@365.univ-ovidius.ro (C.O.); 2 Department of Physical Medicine and Rehabilitation, Faculty of Medicine, “Ovidius” University of Constanta, 1 University Alley, Campus–Corp B, 900470 Constanta, Romania; 3 Faculty of Pharmacy, Ovidius University of Constanta, 1 University Alley, Campus–Corp C, 900470 Constanta, Romania; adina.petcu@365.univ-ovidius.ro; 4 Department of Rehabilitation Medicine, University of Medicine and Pharmacy “Iuliu Hatieganu”, 8 Victor Babes Street, 400012 Cluj-Napoca, Romania; viorela.ciortea@umfcluf.ro; 5 Physical Medicine and Rehabilitation Department, Faculty of Medicine, “Lucian Blaga” University of Sibiu, Victoriei Blvd., 550024 Sibiu, Romania; florina-ligia.popa@ulbsibiu.ro; 6 Department of Dermathology, Faculty of Medicine, “Ovidius” University of Constanta, 1 University Alley, Campus–Corp B, 900470 Constanta, Romania; emma.gheorghe@365.univ-ovidius.ro; 7 Department of Orthopedics and Traumathology, Faculty of Medicine, “Ovidius” University of Constanta, 1 University Alley, Campus–Corp B, 900470 Constanta, Romania; bogdan.obada@365.univ-ovidius.ro

**Keywords:** baker cyst, intermittent vacuum, knee osteoarthritis, ultrasonography, physical therapy, mud, rehabilitation

## Abstract

The Baker cyst (BC), also known as the popliteal cyst or parameniscal cyst, is a fluid-filled sac that normally develops in the back of the knee, between the semimembranosus and medial head of the gastrocnemius. We aimed to evaluate the effectiveness of physiotherapy (10 days of treatment) that associates intermittent vacuum therapy (IVT) on the lower limbs in the treatment of the BC, respectively, in its size reduction. Sixty-five patients with knee osteoarthritis using Kellgren–Lawrence criteria and the presence of BC (ultrasonography evaluation), were divided into the Control and Vacuum groups. We collected the following features: sex, age, level of education, occupation, environment, body mass index, Knee Injury and Osteoarthritis Outcome Score, Western Ontario, and McMaster Universities Osteoarthritis Index, the Functional Independence Measurement, the Fall Risk Score, and the Visual Analog Scale were recorded at baseline and after 10 days. Both groups are similar in terms of demographic features. Regarding the clinical functional parameters, the results elicit a statistically significant change in all parameters between admission and discharge, including the echo volume at BC. Physical medicine and rehabilitation increase the autonomy of patients with BC. Clinical-functional improvement begins in the first 10 days of complex rehabilitation treatment; it is statistically significant and is not different between the two groups, which brings an additional argument for the effectiveness of conservative therapy in the treatment of BC. Although IVT has not demonstrated its superiority over classical balneo-physical therapy, additional research, and long-term monitoring are needed to provide additional arguments regarding this aspect.

## 1. Introduction

Located behind the knee joint, a Baker’s cyst (BC), also known as a popliteal cyst, is a fluid-filled sac. Meniscal tears, rheumatoid arthritis, osteoarthritis (OA), and ligament injuries are among the conditions that frequently result in it. The conservative management of BC includes rest, ice, compression, elevation, physical therapy, pharmaceutical intervention, and, in rare cases, surgical removal. In this study, we sought to evaluate the efficacy of physiotherapy associated with intermittent vacuum therapy (IVT) on the lower limbs in managing the BC and reducing its size, compared to a therapeutic program without intermittent vacuum [1].

The usefulness of physical therapy for treating BC has been examined in several studies. The effectiveness of physical therapy and intra-articular corticosteroid injection in lowering pain and enhancing function in individuals with BC was compared in a randomized controlled trial, and according to the study, both therapies successfully lowered pain and enhanced function, but physical therapy had a more enduring impact than corticosteroid injection [2]. Another study looked at the efficiency of ultrasound-guided aspiration and physical therapy in treating BC. Both therapies were successful in reducing cyst volume, according to the study, but physical therapy had a more robust impact than suction [3].

Pharmacological treatments, such as non-steroidal anti-inflammatory medicines, corticosteroid injections, and physical therapy, can be beneficial in reducing tension and inflammation brought on by BC. According to a systematic study, corticosteroid injections were useful in lowering pain and enhancing function in individuals with BC [4]. An uncommon alternative for treating BC is surgical excision, which is normally only used after all other treatments have failed or when the cyst is seriously causing pain and discomfort. In another study, the results of surgical excision in individuals with BC were examined [5]. The results revealed that surgical excision had a low rate of complications and successfully relieved symptoms in all participants.

Finally, treatment for BC usually entails a combination of conservative management, physical therapy, pharmaceutical therapies, and, in extremely rare circumstances, surgical removal. To create a rehabilitation plan specific to one’s needs and objectives, it is crucial to collaborate with a trained health provider.

IVT, also known as intermittent pneumatic compression, has become increasingly popular in recent years, especially among athletes. The basic hypothesis is that IVT increases blood circulation, thus allowing faster regeneration due to improved exchange of tissue fluids. Intermittent vacuum devices involve various cuffs with inflatable air chambers that are sequentially inflated to generate a peristaltic-like pressure pattern, causing fluids to flow toward the heart [6]. When the intermittent vacuum is used, the limbs are placed in a special chamber where oscillating negative pressure (vacuum) is generated to increase tissue perfusion. IVT increases tissue perfusion and increases oxygen delivery to muscles [7]. Lower limb intermittent negative pressure therapy can improve the skin microcirculation of the quadriceps of male rowers, which has a positive effect on the rapid recovery of physical fitness [8]. Pneumatic compression devices are also applied in clinical practice, for example, in the prevention of venous thromboembolism [9], in wound healing [10], for intermittent claudication [11], or in erectile dysfunction [12]. Therefore, it is essential to provide more evidence on whether compression or vacuum treatment is effective.

IVT may be a viable therapeutic option for BC, according to the mechanisms of action.

We set out to compare a therapeutic program with and without intermittent vacuum therapy on patients to see if intermittent vacuum therapy combined with physiotherapy on the lower limbs is more effective in treating BC and shrinking their size.

## 2. Materials and Methods

### 2.1. Study Model

Between November and December 2022, 65 patients with knee OA and BC were enrolled in this prospective, randomized, controlled, single-blind study at the Balneal and Rehabilitation Sanatorium Techirghiol (BRST), Romania (15 males, 50 females). The Sanatorium Ethical Committee approved the study (approval no. 16,604 from 28 October 2022) and complied with the revised ethical guidelines of the Declaration of Helsinki. The Intermittent Vacuum group (Vacuum) and the Control group (Control) were randomly assigned to two groups of patients (RCT code: NCT06079684).

### 2.2. Study Population

Patients were selected based on the following inclusion criteria: free consent based on the explanation and understanding, respectively, of all related procedural steps; age over 18 years; diagnosis of knee OA with BC. All patients’ osteoarthritis severity was assessed using the Kellgren–Lawrence classification system.

The study excluded patients with neurological and rheumatological conditions, trauma, a history of knee or lower extremity joint surgery, hip or ankle pain or disability, a history of steroid injection in the previous three months, and conditions that would be contraindicated by IVT.

Before starting our work, a study was performed to assess the interobserver reliability of identifying BC and its measurements. Three experts in musculoskeletal ultrasonography independently examined 15 patients to assess the posterior joint compartment, and the examiners were uninformed about clinical data. There was no disagreement between observers about the presence of BC, and the differences between the values of longitudinal, transverse, and anterior-posterior diameters and the structure’s volume were not statistically significant.

Data on demographics and clinical conditions were gathered. We collect the next features: sex, age, level of education, occupation, environment, degree of functional deficit, body mass index (BMI), and Kellgren–Lawrence classification.

Also, scores for the Knee Injury and Osteoarthritis Outcome Score (KOOS), the Western Ontario and McMaster Universities Osteoarthritis Index (WOMAC), the Functional Independence measurement (FIM), the Fall Risk Score, and the Visual Analog Scale (VAS) were recorded at baseline and after 10 days.

**Figure 1:**
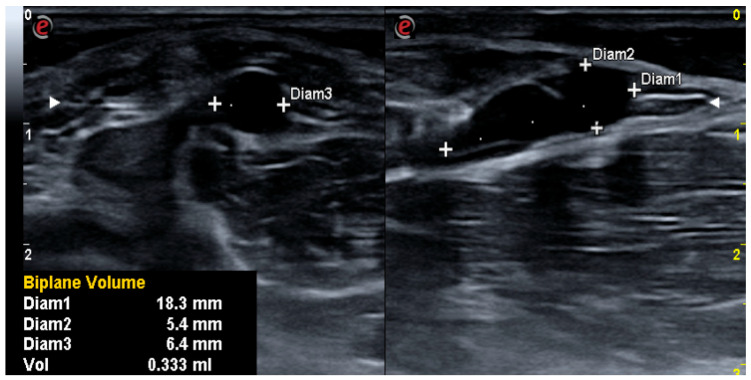
Ultrasonographic images of Baker’s cyst.

A blinded expert used a 7–15 MHz linear probe to take ultrasonographic measurements (Essaote Mylab X6) of BC at baseline and after 10 days. The equipment automatically determined the cyst volume after three measurements were taken in transverse, axial, and longitudinal sections, and the device automatically calculated the cyst’s volume (Figure 1).

### 2.3. Procedures

All groups received a similar physical therapy program, according to the good practice guidelines developed by the Romanian Ministry of Health, according to order 361/2013. Daily, all patients from both groups performed the same program procedures: general warm mud bath (20 min at 38 °C), hydrokinetotherapy in saline water from Lake Techirghiol by a certified physical therapist (20 min at 35 °C), massage therapy for paravertebral muscles, shoulder and pelvis girdle, kinesiotherapy with a standard program for peripheric joints for 30 min.

**Figure 2:**
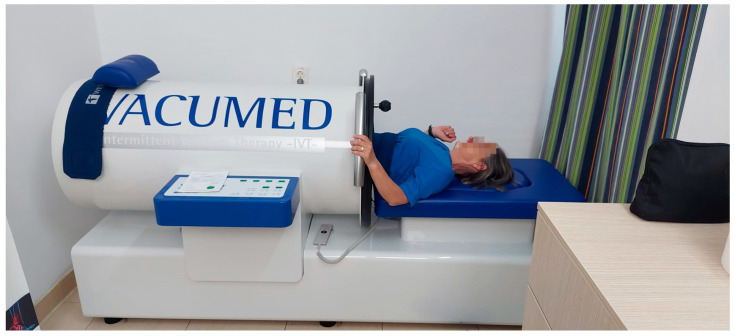
Vacumed device (Weyergans High Care AG)—personal archive.

For Vacuum group, we added to the treatment IVT, performed daily for 30 min, with a Vacumed device (Weyergans High Care AG, Düren, Germany). The device consists of an airtight vacuum chamber and a pump connected to a pressure control system. During vacuum treatments, participants were asked to lay themselves comfortably in a supine position (Figure 2). The lower body was positioned in the vacuum chamber, which was sealed around the participant’s trunk with a cuff at the level of the umbilicus to allow the application of negative pressure. Negative pressure cycles are created by alternating between removing air and venting the chamber to atmospheric pressure.

Patients randomized to the vacuum group received IVT that included a program consisting of 5 successive phases, the duration of which was 6 min each: phase 1 included treatment with an oscillatory protocol of 12 s, followed by a break of 7 s, the pressure being −48 millibars. Phase 2 included treatment with an oscillatory protocol of 15 s, followed by a break of 8 s, the pressure being −50 millibars. Phase 3 included treatment with an oscillating protocol of 21 s, followed by a 9 s break, with the pressure being −56 millibars. Phase 4 included treatment with an oscillatory protocol of 19 s, followed by a break of 8 s, the pressure being −60 millibars. Phase 5 included treatment with an oscillatory protocol of 15 s, followed by a 7 s break, with the pressure being −50 millibars.

### 2.4. Randomization, Group Allocation and Blinding

All patients were recruited at the BRST by a clinician blinded as to group allocation. Each study group was composed through random allocation of the total sample with a 1:1 allocation ratio that was reported to patients by a different researcher, who was neither the one who performed the treatment nor the one who performed the evaluation, through a pre-specified allocation list who was concealed in a password-protected computer file.

### 2.5. Sample Size

Of the total of 102 patients who were presented for hospitalization in the BRST between November and December 2022, only 65 completed the participation conditions, being grouped into two groups of 33 and 32, respectively. Due to the relatively small number of patients, the statistical approach was a non-parametric one: Independent Samples Mann–Whitney U test, Independent Sample Median Test, Reb test Samples Wilcoxon Signed Rank test, and Chi-Square test.

### 2.6. Study Objectives

The first aim of the study was to conduct a comparison between Vacuum and Control patients regarding changes in volume BC after 10 days of a complex rehabilitation program. The second aim of this study is to determine if there are clinical-functional benefits after 10 days of rehabilitation treatment in patients with knee OA and BC.

### 2.7. Data Analysis

The statistical analysis was performed using IBM SPSS statistics software version 23. Data are presented as mean ± standard deviation (SD) for continuous variables in cases of symmetric distributions, median and IQR (Interquartile range IQR = P75–P25) for numerical discrete variables or for continuous variables in cases of skewed distributions, or as frequencies and percentages for categorical variables. The normality of the continuous data was estimated with Shapiro–Wilk Tests of Normality. For hypotheses testing: Independent Samples Mann–Whitney U test, Independent Samples Median test, Related Samples Wilcoxon Signed Rank Test, Chi-Square Test of association were used depending on the type of analyzed variables. The significance level α was set at 0.05. If the test statistic for every test conducted was in the critical region and the *p*-value was less than or equal to the significance level, we decided to reject the null hypothesis in favor of the alternative hypothesis.

## 3. Results

### 3.1. Study Population

**Figure 3:**
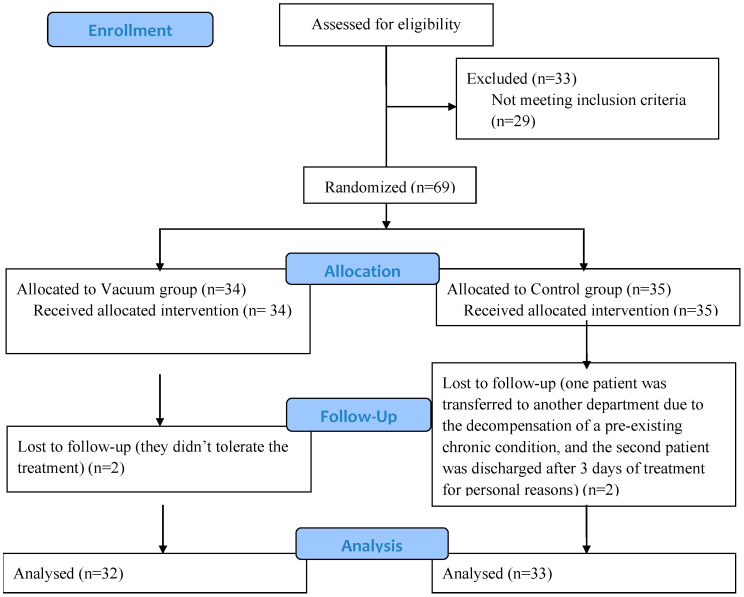
Flowchart of selection of the participants.

A total of 102 patients were invited to the study. This study was conducted on 65 patients with knee OA and BC (Figure 3) and was divided into a Vacuum group with 32 patients and a Control group with 33 patients.

Demographic characteristics are presented in Table 1. The average age of the Vacuum patients was 58.66 years, and for the Control patients, it was 61.36 years. Patients from the Control group were older than patients from the Vacuum group (87.88% of the Control group were more than 50 years old). In the Vacuum group, 10 (31.25%) of the patients were males and 22 (68.75%) were females. In the Control group, 5 (15.15%) of the patients were males, and 28 (84.85%) of them were females. Most of the patients are from urban environments: 50 (76.92%) from Vacuum and 15 (23.08%) from Control. When the patients were evaluated based on occupation/profession, we found that the Vacuum group consisted of 15 working individuals, 14 retired individuals, and 3 unemployed, whereas for the patients in the Control group, we found 11 working individuals, 20 retired individuals, and 2 unemployed. When the two groups were compared, the percentage of patients who were retired represented the majority in both groups (Vacuum 43.75% and Control 60.61%). Analyzing the BMI, we observed similar values in the two groups, with no statistically significant differences between them (*p* = 0.840 > 0.05). After taking the radiographs, the degree of knee OA was determined using the Kellgren–Lawrence classification, and thus, we observed similarities between the groups, the differences not being statistically significant (*p* = 0932 > 0.05).

So, there is no statistically significant association between the two groups regarding demographic features: sex, age, level of education, occupation, environment, BMI, and Kellgren–Lawrence classification, both groups being similar.

### 3.2. Clinical–Functional Status

**Figure 4:**
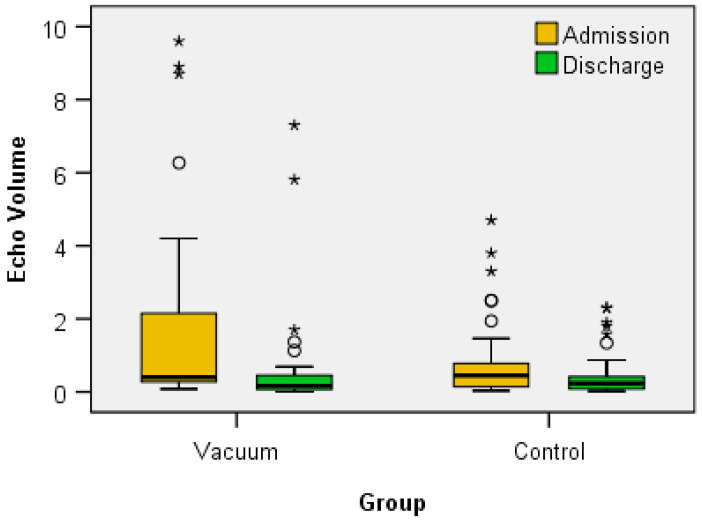
The variation of BC sizes at admission/discharge in the two groups.

Analyzing the echo volume at BC (Figure 4), the Wilcoxon signed-rank test showed that for both the Vacuum group (*Z* = −4.469, *p* < 0.001) and the Control group (*Z* = −2.921, *p* = 0.003), the treatment elicits a statistically significant change in the dimensions between admission and discharge (Echo Volume at discharge < Echo Volume at admission).

Also, we analyzed the structure of BC, and there was no change in echogenicity, the presence of septa or intracystic bodies. Likewise, no BC ruptures were reported.

Regarding the functional status, we analyzed the evolution of the fall risk, VAS, total FIM score, WOMAC, KOOS (symptoms, pain, daily activities—ADL, sports and recreational activities, quality of life—QOL and total), and echo volume of BC at admission and discharge (Table 2 and Table 3).

**Figure 5:**
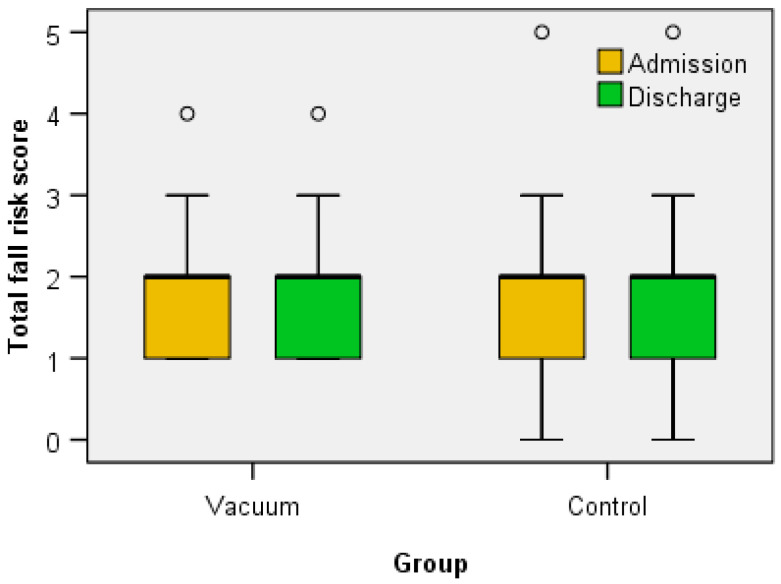
The variation of total fall risk score between admission and discharge.

The Mann–Whitney U Test revealed no significant differences in the Total fall risk score at admission between the Vacuum group (Median = 2.00, n = 32, Mean rank = 34.39) and the Control group (Median = 2.00, n = 33, Mean rank = 31.65), U = 483.5, z = −0.650, *p* = 0.516 > α = 0.05, and also no significant differences in the Total fall risk score at discharge between the Vacuum group (Median = 2.00, n = 32, Mean rank = 34.11) and the Control group (Median = 2.00, n = 33, Mean rank = 31.92), U = 492.5, z = −0.532, *p* = 0.601 > α = 0.05, as shown in Table 2 and Figure 5.

A Wilcoxon signed-rank test showed that for both the Vacuum group (Z = −1.000, *p* = 0.317) and the Control group (Z = 0.000, *p* = 0.999), the treatment did not elicit a statistically significant change (Table 2 and Figure 5) in the Total fall risk score between admission and discharge.

**Figure 6:**
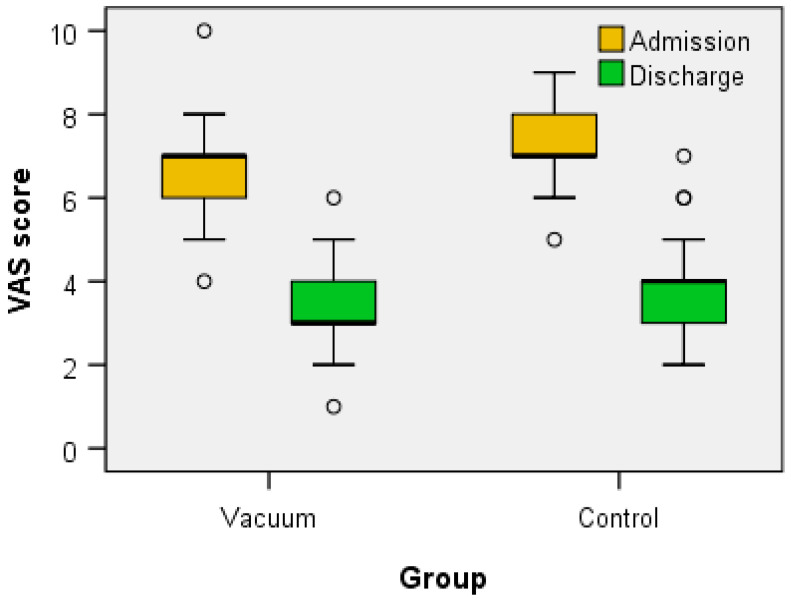
The variation of VAS score between admission and discharge.

**Figure 7:**
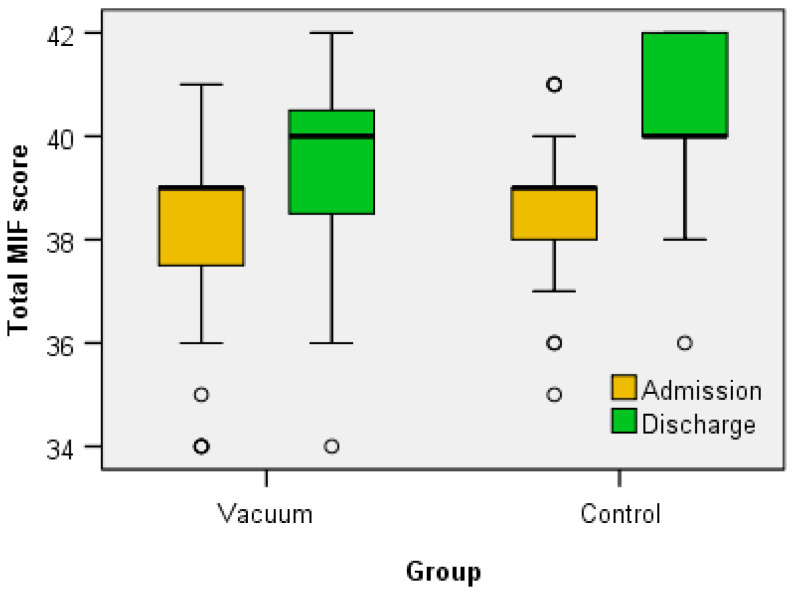
The variation of MIF score between admission and discharge.

**Figure 8:**
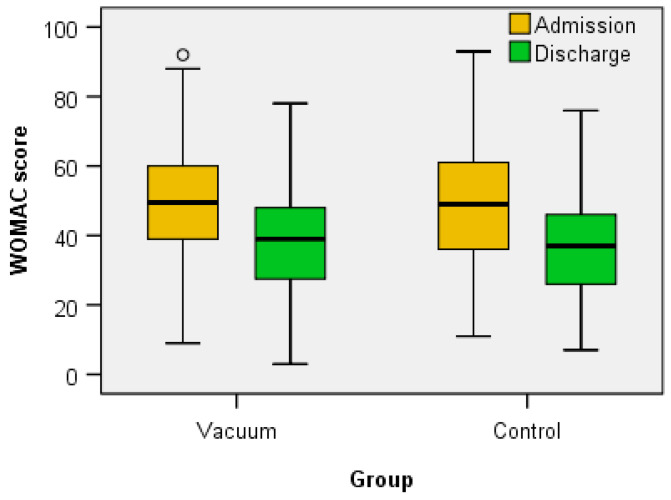
The variation of WOMAC score between admission and discharge.

**Figure 9:**
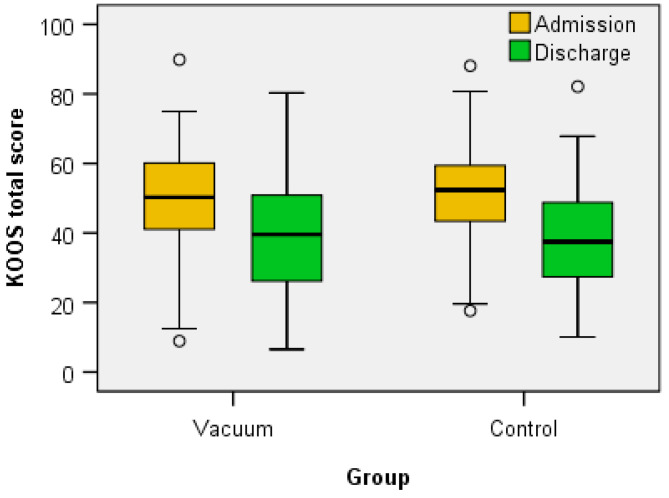
The variation of total KOOS score between admission and discharge.

Regarding the clinical functional parameters analyzed (VAS score, total FIM score, WOMAC score, KOOS (symptoms, pain, daily activities—ADL, sports and recreational activities, quality of life—QOL and total)), Wilcoxon signed-rank test showed that for both the Vacuum group and the Control group the treatment elicits a statistically significant change in all parameters (Table 2 and Table 3 and Figure 6, Figure 7, Figure 8 and Figure 9) between admission and discharge (score at discharge < score at admission, except FIM score that increases at discharge).

## 4. Discussion

Rehabilitation in knee OA with BC is essential to improve knee function and mobility and to reduce pain and inflammation. There are different ways to rehabilitate, which may include physical therapy, muscle-strengthening exercises, lifestyle changes, and surgery, depending on the severity of the condition. The environment and people’s behavior can also affect the way rehabilitation works. For instance, the facility operated by the BRST in a region near the Black Sea is known for providing effective and comprehensive care to injured individuals. The region’s natural cure factors have an impact on the services and locations of the facility.

Here, salty mineral waters, therapeutic muds and other spa treatments are used for various ailments of the musculoskeletal, rheumatic, dermatological, respiratory, and endocrine systems.

The mineral waters of Techirghiol are recognized for their therapeutic properties, the high content of mineral salts, among which are sodium chloride, bromide, and iodide, as well as trace elements such as magnesium, calcium, iron, and manganese. Techirghiol therapeutic muds are known for their anti-inflammatory and analgesic properties and are used in the treatment of rheumatic, peripheral nervous system, immune, and respiratory conditions [13,14,15].

Our hospital’s daily hydrokinetic therapy program consists of a hot saline/mud bath, which is the only major procedure that involves the use of hydrokinetic energy. Other procedures, such as electrotherapy, ultrasound therapy, and massage therapy, are also commonly utilized [16].

There are many studies that have evaluated the effectiveness of physical therapy in the treatment of knee OA and BC. The results showed that physical therapy can reduce pain and inflammation, improve knee mobility, and function, and reduce the size of the BC [17]. Another study examined the effectiveness of muscle-strengthening exercises in knee OA and BC. The results showed that muscle-strengthening exercises can improve muscle strength and knee function, reduce pain and inflammation, and improve patients’ quality of life [18].

In addition, lifestyle changes, such as losing weight and avoiding activities that put pressure on the knee, can help reduce pain and inflammation.

In our study, the over-50 age category predominates in both groups, as does the prevalence of BC in women. Analyzing the literature and estimating the exact incidence and prevalence of BC in the general population can be difficult, as this condition can be asymptomatic and may be diagnosed incidentally during a medical examination of the knee.

A study published in the journal Clinical Rheumatology examined the incidence of BC in patients with rheumatoid arthritis and found that BC was present in approximately 30% of these patients [19]. Additionally, an imaging study of a group of healthy individuals found that approximately 20% of these individuals had popliteal cysts [20]. However, in general, there are no accurate data on the incidence and prevalence of BC in the general population. It is important to recognize that BC is a benign condition that can be successfully treated, if necessary, with the help of a physical medicine and rehabilitation specialist or an orthopedic surgery specialist.

There are some studies that suggest that BC is more common in women than in men, but there are not yet enough data to categorically confirm this association. A study examined the incidence of BC in patients with rheumatoid arthritis and found that the condition was more common in women than in men [19]. Another study also found that BC was more common in women in the over-50 age group [21]. However, it is important to note that these studies are limited, and that further research is needed to confirm the association between BC and patient gender.

From the point of view of the demographics of the patients in our study, we found that the majority are from the urban environment and are employed or retired. There are insufficient data available to suggest an association between the presence of BC and the rural/urban environment or occupation/physical activity. Although there are certain conditions or physical activities that can increase the risk of developing a BC, such as osteoarthritis or trauma to the knee, these are not directly related to the environment or profession. However, it is important to note that this condition can occur in any person, regardless of gender, age, background, or profession. Risk factors that may increase the chances of developing a BC include a history of knee injury, arthritis, obesity, and advanced age [21].

In our study, both in the Vacuum group (mean BMI = 29.00) and in the Control group (mean BMI = 28.78), the overweight class predominates, which is also in accordance with the specialized literature that says there is an association between BMI and the presence of BC. Studies have shown that people who are overweight or obese have a higher risk of developing BC compared to people of normal weight. A study published in the Skeletal Radiology Journal found that people with a higher BMI had a higher risk of developing BC. In addition, this study showed that overweight or obese patients experienced a greater degree of pain and limitation of knee function associated with BC [22]. Another study found that people with a higher BMI had a significantly higher risk of developing BC. Also, this study showed that individuals with a higher BMI and BC had significantly greater limitations in knee function compared to individuals with a lower BMI and BC [23].

The radiological evaluation of the patients in both groups places the majority of them in Kellgren–Lawrence grade 3, which agrees with the specialized literature that says there is a link between the Kellgren–Lawrence classification and the presence of BC. A study showed that there is an association between the advanced stage of OA, according to the Kellgren–Lawrence classification, and the prevalence of BC. Specifically, the prevalence rate of BC was higher in patients with more advanced stages of OA [24]. Also, another study showed that there is a significant correlation between the size of the BC and the stage of OA according to the Kellgren–Lawrence classification. This study found that BC is more common in patients with advanced stages of OA and that cyst size is directly proportional to the severity of OA [25].

The first objective of our study, namely, the evaluation of the dimensions of the BC under the effect of the recuperative balneal-physical-kinetic treatment, showed that, indeed, the volume decreases statistically significantly in both analyzed groups, IVT not being statistically superior to classical therapy, although the decrease in BC size was greater than classical therapy, but without statistical significance.

It should be mentioned that this study was conducted for a duration of only 10 days, while numerous studies conducted by other researchers were conducted for longer periods. For example, a study evaluated the effects of a therapeutic exercise program on patients with knee OA. The results showed a significant improvement in knee function and patients’ quality of life [26]. Also, another study evaluated the effects of an 8-week physiotherapy program on patients with BC and knee OA. Results showed a significant decrease in BC size and a significant improvement in knee function and knee pain after exercise therapy [27]. Therefore, it should be mentioned that in our study, remarkable results were obtained in a shorter period, which could justify the benefits that the balneal-climatic factors of the Techirghiol area (sapropelic mud and mineral salty water of the lake) have in amplifying the effects of physical-kinetic therapy.

There are also studies suggesting that spa treatment, which includes peloid therapy and mineral water, may be helpful in the treatment of BC and associated knee OA. However, it is important to keep in mind that these studies are relatively small, and more research is needed to confirm these results and identify the mechanisms by which spa treatment may be beneficial [28,29,30]. There are other studies that investigated the effect of physiotherapy on BC and evaluated the reduction in its size [31,32,33].

Researching the international databases regarding the change in size of the BC depending on various factors, we conclude that there are not much data regarding this aspect. It seems that there is evidence that an increase in chondral lesion severity increases the amount of effusion and cyst volume, the degree of chondral lesion was moderately and positively correlated with cyst volume in the total population, and the degree of the chondral lesion was moderately and positively correlated with the degree of effusion [25]. At the same time, cartilage degeneration, medial plicae, and an increase in intra-articular effusion may increase the BC volume [34]. Another study evaluated the effectiveness of galvanic current and dexamethasone iontophoresis in the treatment of knee OA and BC, and the results show significant clinical and functional improvement was detected with dexamethasone iontophoresis in the treatment of patients with knee OA and BC [35]. We did not find any other information in the previously conducted research about the evolution of BC in knee OA during medical rehabilitation treatment.

All the patients included in this study were assessed for their risk of falling, and it was found that the majority obtained 2 points, which placed them in the category of those with a low risk of falling. In the literature, there are studies that show a possibility that the presence of a BC increases the risk of falls in patients with OA of the knee, but there is not enough research to confirm this relationship. However, knee OA, which can be associated with BC, is a condition that can increase the risk of falls in the elderly. For patients with OA of the knee, pain and stiffness in the joint can lead to reduced mobility and a limited ability to move, which can increase the risk of falling [36]. Also, it was found that patients with BC and knee OA had lower physical function and reduced mobility compared to patients with knee osteoarthritis without BC. This could increase the risk of falls in these patients [37].

At the same time, the clinical-functional evaluation of the patients included the evaluation of pain using the VAS scale. Most of the patients self-assessed their pain with a score of 7 at admission, both those in the Vacuum group and those in the Control group. After 10 days of balneal-physical-kinetic treatment, remarkable results were obtained, with a greater decrease in the pain score in the Vacuum group (value 3), compared to the Control group (value 4). There are studies that have investigated the relationship between the VAS score and the presence of BC in patients with knee OA. It is generally believed that the presence of BC may be associated with a higher level of knee pain, but there is no clear consensus on this relationship [26,37,38,39,40].

A study published in the American Journal of Physical Medicine and Rehabilitation found that patients with a BC had higher VAS scores than patients without a BC, suggesting a possible association between a BC and the level of knee pain [39]. In another study, it was found that there is a positive correlation between the size of the BC and the level of knee pain. However, research has shown that there is no correlation between the presence of a BC and the severity of knee OA symptoms [34].

Considering the FIM score, the patients in both groups were included in the class of those with a minimal functional deficit, both cognitively and motorically, and comparing the two groups, there were no statistically significant variations. In general, in specialized literature, there is no direct link between the FIM score and the presence of BC in patients with knee OA. However, it is possible that the presence of the BC and other symptoms of knee OA may affect patients’ quality of life and thus influence the FIM score. Another study found that patients with BC and knee OA had a lower level of quality of life compared to patients with knee OA without BC. The researchers also found that the size of BC was associated with a lower level of quality of life in patients with knee OA [1,26].

The WOMAC score, used to evaluate the severity of symptoms and functionality in patients with knee OA, on a scale from 0 to 96, recorded average values of 48.53 in the Vacuum group and 51.06 in the Control group, and analyzing the dynamic values, we found a statistically significant decrease between admission and discharge scores in both groups. There are studies that evaluated the relationship between the WOMAC score and the presence of BC in patients with knee OA [26]. Another study found that the presence of BC was associated with a higher WOMAC score in patients with knee OA. The researchers concluded that the presence of BC may worsen symptoms and dysfunction in patients with knee OA [41]. Another study found that the presence of a BC was associated with a higher WOMAC score in patients with knee OA and, therefore, lower functionality [27].

Regarding the effectiveness of a rehabilitation program for patients with BC, a study evaluated it and found that it resulted in a significant improvement in knee function and pain reduction [42].

The KOOS questionnaire is a symptom and function assessment tool for patients with knee conditions such as OA. The questionnaire is composed of 42 items that cover 5 different domains: pain, symptoms, functionality–activities of daily life, functionality–sports and recreation and quality of life. The total questionnaire score is calculated by adding the scores for each domain and converting them to a score from 0 to 100, where 0 represents the worst functionality, and 100 represents the best functionality [43]. Carrying out this questionnaire on all the patients included in the study, we found that there were statistically significant decreases in all the values of the analyzed items at admission/discharge in both groups. There is research suggesting that physiotherapy and exercise may be beneficial in the treatment of BC in patients with OA of the knee and may improve KOOS assessment results [44,45].

### 4.1. Study Strengths

As a strong point, we can say that in the international databases, we have not found any study that evaluates intermittent vacuum therapy in reducing BC sizes. The patients completed a thorough course of treatment that included physical therapy and balneary components (lake saltwater treatment). Due to the dearth of information in the literature, numerous investigations are required in this field. Another asset is the study’s overall patient enrollment, which is representative and evenly split between the two groups. The article was written and created in accordance with ethical standards, considering the deontological implications of disseminating scientific findings. Another strong point of this study is the complex clinical-functional and imaging evaluation of relatively large groups of patients, which provided important details about the effectiveness of balneal-physical treatments.

### 4.2. Study Limitations

This study has several limitations. First, it is necessary to monitor the long-term evolution, both from the clinical-functional and imaging points of view, of BC dimensions. The dynamics data indicate that the clinical-functional parameters have improved after only 10 days of treatment, although further treatment or follow-up appointments may be required. VAS, which is a subjective judgment, was unable to provide us with precise information regarding the sort of pain.

### 4.3. Facts and Perspectives

In general, it is important to treat BC as soon as possible to prevent the progression of the condition and improve knee function. In addition, physical and balneal therapy can help relieve symptoms associated with a BC, such as pain and stiffness, and improve joint function. Mud baths, salty baths, stretching exercises, muscle training, and other physical therapy techniques can be adapted to help reduce the inflammation and discomfort associated with BC. It is important to consult with a physical therapy specialist to determine the best treatment plan based on the size of the cyst and the patient’s general condition.

## 5. Conclusions

Physical medicine and rehabilitation increase the autonomy of patients with BC. Clinical-functional improvement begins in the first 10 days of complex rehabilitation treatment; it is statistically significant and is not different between the two groups, which brings an additional argument for the effectiveness of conservative therapy in the treatment of BC. Although intermittent vacuum therapy has not demonstrated its superiority over classical balneo-physical therapy, additional research and long-term monitoring are needed to provide additional arguments regarding this aspect.

## Figures and Tables

**Table 1:** Patients’ demographic and clinical characteristics.

	Vacuum						Control						*p*
	n	%	Mean	Median	Min–Max	Std Dev	n	%	Mean	Median	Min–Max	Std Dev	
Sex													0.124
Male	10	31.25					5	15.15					
Female	22	68.75					28	84.85					
Age (years)			58.66		43–77	8.71			61.36		43–75	8.34	0.181
Studies													0.343
High school	2	6.25					0	0					
College	2	6.25					2	6.06					
Unspecified	28	87.5					31	93.94					
Occupation													0.395
No occupation	3	9.38					2	6.06					
Employee	15	46.88					11	33.33					
Retired	14	43.75					20	60.61					
Environment													0.415
Urban	26	81.25					24	72.73					
Rural	6	18.75					9	27.27					
Degree of functional deficit													0.120
Minimum	26	81.25					31	93.94					
Moderate	6	18.75					2	6.06					
BMI (kg/m^2^)			29	28.58	22.99–42.9	4.67			28.78	27.92	21.97–38.28	3.98	0.840
Kellgren–Lawrence			2.56	3	1–4	0.91			2.61	3	1–4	0.93	0.932

**Table 2:** Clinical–functional status of VACUUM/CONTROL: intergroup differences in the temporal changes of Fall risk score, VAS, FIM, WOMAC, KOOS, Echo volume.

	Vacuum					Control					*p*
	Mean	Median	Std Dev	Min–Max	Percentiles 25–75	Mean	Median	Std Dev	Min–Max	Percentiles 25–75	
Fall risk score											
Admission	1.78	2	0.71	1–4	1–2	1.75	2	0.67	1–4	1–2	0.516
Discharge	1.75	2	0.67	1–4	1–2	1.7	2	0.88	0.00–5	1–2	0.601
p admission/discharge	0.317					0.999					
VAS scale											
Admission	6.66	7	1.04	4–10	6–7	7.15	7	0.76	5–9	7–8	0.009
Discharge	3.31	3	1.09	1–6	3–4	3.88	4	1.08	2–7	3–4	0.032
p admission/discharge	0.009					0.032					
FIM score											
Admission	38.09	39	1.97	34–41	37.25–39	38.67	39	1.29	35–41	38–39	0.474
Discharge	39.41	40	1.83	34–42	38.25–40.75	40.21	40	1.43	36–42	40–42	0.077
p admission/discharge	0.000					0.000					
WOMAC											
Admission	48.53	49.5	19.58	9–92	38.5–60	51.06	49	20.45	11–93	35.5–64.5	0.844
Discharge	37.28	39	19.18	3–78	27.25–48	37.03	37	16.4	7–76	25.5–47	0.718
p admission/discharge	0.000					0.000					
KOOS total											
Admission	47.85	50.27	19.98	8.91–89.86	39.86–60.39	51.46	52.35	17.35	17.63–88.07	43.43–60.34	0.679
Discharge	37.63	39.56	18.54	6.54–80.33	24.38–51.01	38.6	37.47	15.66	10.10–82.11	27.06–49.08	0.990
p admission/discharge	0.000					0.000					
Echo volume											
Admission	1.83	0.40	2.77	0.08–9.6	0.26–2.47	0.88	0.45	1.19	0.03–4.7	0.12–0.84	0.205
Discharge	0.70	0.17	1.6	0.00–7.3	0.07–0.48	0.51	0.23	0.69	0.02–2.3	0.08–0.55	0.599
p admission/discharge	0.000					0.003					

**Table 3:** KOOS SCORE (divided by its 5 items: symptoms, pain, ADL, sports, QoL).

	Vacuum					Control					
	Mean	Median	Std Dev	Min–Max	Percentiles 25–75	Mean	Median	Std Dev	Min–Max	Percentiles 25–75	*p*
KOOS symptoms											
Admission	7.36	7.44	3.28	1.78–15.47	4.76–9.81	8.14	8.92	3.54	2.38–14.8	4.46–10.71	0.357
Discharge	5.91	5.95	3.44	0.5–14.88	2.97–3.87	6.39	6.54	2.89	0.59–13.09	3.87–8.3	0.430
p admission/discharge	0.000					0.000					
KOOS pain											
Admission	9.52	10.12	4.57	1.78–19.04	5.21–12.5	10.2	11.3	3.98	3.57–19.04	7.14–12.8	0.708
Discharge	7.19	8.33	3.85	0.00–16.07	3.72–9.96	7.53	7.14	3.66	0.59–13.09	5.3–10.11	0.849
p admission/discharge	0.000					0.000					
KOOS ADL											
Admission	17.45	19.04	9.12	1.19–9.52	4.91–10.56	19.26	20.23	7.67	4.76–38.05	14.28–23.21	0.390
Discharge	13.24	13.99	7.74	0.00–11.9	3.13–8.77	14.13	13.69	6.92	2.38–35.71	9.52–18.45	0.769
p admission/discharge	0.000					0.000					
KOOS sports											
Admission	7.68	8.03	3.25	1.19–11.9	4.91–10.56	7.89	8.92	2.77	1.78–11.9	6.25–9.22	0.911
Discharge	6.17	6.54	3.48	0.00–11.9	3.13–8.77	5.89	5.8	2.47	1.19–11.9	4.46–8.03	0.631
p admission/discharge	0.000					0.000					
KOOS QoL											
Admission	5.76	5.65	2.22	1.19–11.9	4.91–10.56	5.9	5.95	1.62	2.3–8.92	5.35–7.14	0.900
Discharge	5.28	5.95	1.62	0.00–11.9	3.13–8.77	4.8	4.7	1.76	2.3–8.92	3.57–5.65	0.228
p admission/discharge	0.005					0.001					

## Data Availability

Data can be found in description of the study registrated RCT code: NCT06079684.
